# Melting Behavior of Compression Molded Poly(ester amide) from 2,5-Furandicarboxylic Acid

**DOI:** 10.3390/polym16243459

**Published:** 2024-12-11

**Authors:** Enrico Bianchi, Michelina Soccio, Massimo Gazzano, Lazaros Papadopoulos, Tobias Robert, Dimitrios N. Bikiaris, Nadia Lotti

**Affiliations:** 1 Department of Civil, Chemical, Environmental and Materials Engineering, University of Bologna, Via Terracini 28, 40131 Bologna, Italy; enricobianchi1994@gmail.com (E.B.); nadia.lotti@unibo.it (N.L.); 2 Interdepartmental Center for Industrial Research on Advanced Applications in Mechanical Engineering and Materials Technology, CIRI-MAM, Viale del Risorgimento 2, 40136 Bologna, Italy; 3 Interdepartmental Center for Industrial Research on Buildings and Construction, CIRI-EC, Via del Lazzaretto 15/5, 40131 Bologna, Italy; 4 Synthesis and Photoreactivity Institute, CNR, Via Gobetti 101, 40129 Bologna, Italy; massimo.gazzano@isof.cnr.it; 5 Laboratory of Polymer Chemistry and Technology, Department of Chemistry, Aristotle University of Thessaloniki, 54124 Thessaloniki, Greece; lazaros.geo.papadopoulos@gmail.com (L.P.); dbic@chem.auth.gr (D.N.B.); 6 Fraunhofer Institute for Wood Research, Wilhelm-Klauditz-Institut, 38108 Braunschweig, Germany; tobias.robert@wki.fraunhofer.de; 7 Interdepartmental Center for Agro-Food Research, CIRI-AGRO, Via Quinto Bucci 336, 47521 Cesena, Italy

**Keywords:** thermal properties, WAXS, melting behavior, FDCA, polyester, food packaging, polymeric film

## Abstract

PEA 46 is a biobased polymer with promising properties for sustainable packaging applications, which can be obtained via polymerization of a furan 2,5-dicarboxylic acid (2,5-FDCA) derivative and a diol monomer containing internal amide bonds (46 amido diol). In the literature, PEA 46 showed a complex series of thermal transitions during DSC scans. For this reason, in this initial exploratory study PEA 46 was subjected to compression molding and the melting behavior of film samples was investigated with parallel DSC and WAXS analyses. At room temperature, a mesomorph phase was the only one observed. Subjecting the samples to heating scans led to the formation of phase α, caused by a sequence of partially overlapping melting and recrystallization phenomena. An additional melting and recrystallization phenomenon resulted in the development of a phase β, which melted at approximately 173 °C, the temperature after which the material was completely amorphous and isotropic. Phase α could be enhanced via thermal annealing, whereas phase β could be enhanced via a melt crystallization treatment.

## 1. Introduction

In recent years, the demand for materials with a reduced environmental footprint has significantly increased [1]. The reason behind it is the growing awareness regarding the depletion of fossil-based resources, fluctuation of oil prices and the detrimental impacts of plastics pollution on ecosystems and human health [2]. As a result, research efforts have been dedicated on the utilization of biobased precursors for polymer synthesis, to develop more sustainable alternatives to commercially available plastics [3]. Among those, 2,5-furandicarboxylic acid (2,5-FDCA) has drawn special attention. It is a monomer of huge potential, as highlighted by its inclusion in the top 12 biobased monomers list that was issued by the US Department of Energy [4]. More importantly, polyesters derived from FDCA are considered as potential replacements to their terephthalic analogues that currently dominate the market, as they provide a unique combination of properties, including excellent mechanical strength, thermal stability and gas barrier performance [5].

One significant step of the evaluation of furan-based materials as substituents for terephthalic acid-based ones in injection molding or fiber applications is the comprehensive understanding of their thermal properties, particularly the crystallization process and melting behavior. The crystallinity of polymeric materials is a crucial aspect of their microstructure, since it influences several key functional properties, including mechanical properties, dimensional and thermal stability and gas permeability [6,7]. Thus, understanding crystallization conditions is essential for the design of industrial processes, to produce materials suitable for targeted applications [8]. Existing studies in the literature often focus on furan-based polyesters with diols of different chain length. Homopolymers with a shorter diol length often display slower crystallization kinetics compared to their terephthalic counterparts, while increasing the diol length facilitates chain orientation and results in faster crystallization [9,10,11,12,13,14,15,16,17,18]. Studies have also shown the existence of a partially ordered 2D-phase, formed as a result of intermolecular hydrogen bonding, and often in competition with 3D-ordered phases [19,20].

Polyamides and poly(ester amide)s (PEAs) derived from aromatic building blocks are another class of polymers of high interest. These materials usually exhibit superior thermomechanical performance as a result of their high crystallinity, which is derived from intermolecular hydrogen bonding [21,22,23]. In FDCA-based polyamides and poly(ester amide)s, intramolecular hydrogen bonds occur, preventing intermolecular hydrogen bonds and resulting in amorphous materials with low degrees of crystallinity [24,25]. Recently, it was possible to demonstrate a new synthetic pathway to circumvent this limitation, by employing pre-formed amido diols, with internal amide bonds and long aliphatic spacers between the furan ring and the amide bonds [26,27]. The functional properties of the synthesized materials were also studied, showing that they have excellent mechanical and gas barrier properties, making them a suitable candidate for sustainable food packaging applications. For example, the copolymerization of PEAs with poly(decamethylene furanoate) (PDF), resulted in a decrease up to about 50% of O_2_ and CO_2_ transmission rates, leading to gas barrier properties comparable to those of commercial poly(ethylene terephthalate) (PET). Also, in terms of mechanical properties, PDF copolymers with PEAs had increased toughness and elongation at break up to 650% [28]. However, these materials displayed a rather complex thermal profile comprised of multiple overlapping phenomena. This finding was particularly interesting considering that polyamides are known to undergo complex crystal-to-crystal transitions, such as the Brill transition [29]. In general, the knowledge of the thermal properties of polymeric materials is fundamental, since thermal properties determine the range of temperatures in which processing can happen, and influence the functional properties of a polymer at a specific temperature. Specifically, the knowledge of the thermal properties of polymers for food packaging applications is critical because thermal transitions determine the material’s suitability for specific conditions of transportation and storage, thus influencing the shelf life of food products. Understanding the thermal properties of polymeric materials for food packaging is a powerful and necessary tool for their improvement, which can lead to reductions in food loss and food waste [30,31].

For all the reasons discussed, this work was intended as an initial exploratory study with the objective of gaining a deeper understanding on the thermal and structural transitions found in PEA 46. The work was focused on polymeric compression-molded samples, since they simulate industrially produced films, and for this reason they can give specific information on the properties of the studied material for practical use in applications such as food packaging. Homopolymer films of poly(ester amide) 46 (PEA 46) were prepared by compression molding, then subjected to Wide Angle X-ray Scattering (WAXS) experiments at different temperatures and during heating or cooling scans. Drawing inspiration from the literature [32,33], Differential Scanning Calorimetry (DSC) experiments were carried out asynchronously with respect to WAXS analyses, but under the same conditions, allowing the establishment of thermal-structural correlations.

## 2. Materials and Methods

### 2.1. Materials and Sample Preparation

The PEA 46 sample used for this work was extensively characterized and studied in the literature [27,28] and had an intrinsic viscosity of 0.38 dL/g. The preparation of the compression molded sample used a C12 laboratory press (Carver, Wabash, IN, USA). About 2 g of polymer was compressed between two Teflon sheets, applying a pressure of 5 ton/m^2^ and maintaining a temperature 30 °C above the highest melting point. The film was approximately 0.3 mm thick and was cooled at room temperature, then stored in a desiccator for 2 weeks before analyses.

### 2.2. Thermal Characterization

A DSC6 (PerkinElmer, Waltham, MA, USA) was utilized for the purpose of conducting Differential Scanning Calorimetry (DSC). The procedure involved the measurement of approximately 5 mg of the sample, which was then placed in an aluminum pan. Subsequently, the pan was sealed with a lid and inserted into the calorimeter. The sample was then subjected to a heating or cooling scan under nitrogen flow of 20 mL/min. The heating rate was set at 20 °C/min, unless otherwise specified. The glass transition temperature (T_g_) was determined by determining the midpoint of the change in baseline of the calorimetric curve, and its specific heat increment (Δc_p_) was calculated based on the difference between the first and second baseline. Furthermore, the melting temperature (T_m_) was identified by locating the maximum of the endothermic peaks, and the melting enthalpy (ΔH_m_) was determined by calculating the area between the peak and the baseline.

### 2.3. Diffractometric Characterization

Wide-angle X-ray Scattering (WAXS) analyses were conducted using an X’PertPro diffractometer (PANalytical, Almelo, The Netherlands), equipped with a solid-state X’Celerator detector. The settings for the analysis included a movement rate of 100 s/step, with a step of 0.1°, and the X-ray source used was copper with a wavelength of 0.154 nm. Non-simultaneous WAXS experiments were also carried out during a temperature scan, at various heating or cooling rates, with a TTK450 device (Anton Paar, Gratz, Austria). The temperature scan approximated DSC ramps, which had been performed separately. At chosen temperature values, the temperature scan was interrupted, so that a WAXS scan could be performed. The degree of crystallinity (X_c_) was calculated without considering incoherent scattering, by subtracting the amorphous halo from the crystalline reflections, then dividing them by the total area of the diffraction curve.

## 3. Results and Discussion

### 3.1. Preliminary Investigation

**Figure 1:**
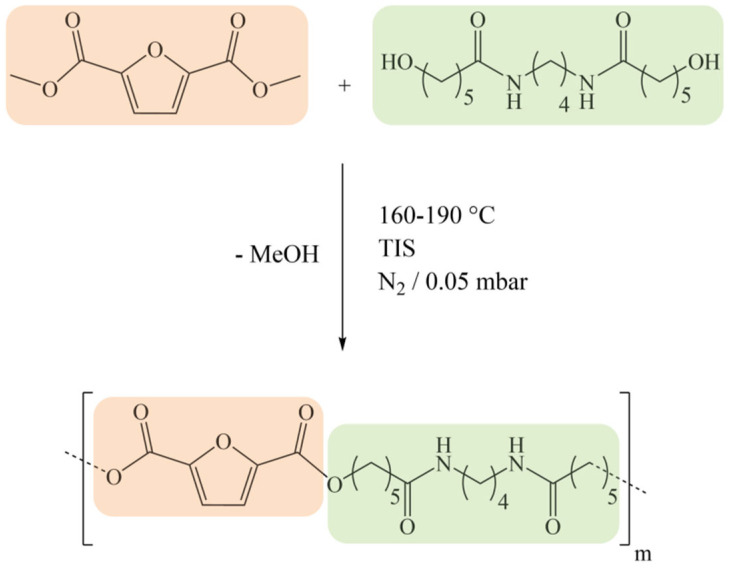
Synthesis and structure of PEA 46 [27].

**Figure 2:**
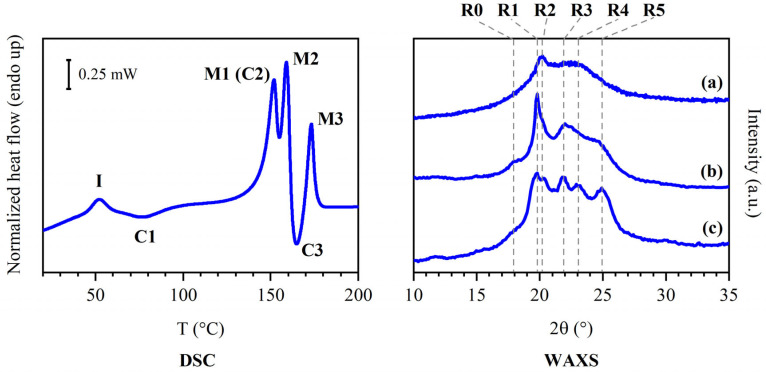
Assignment of labels to DSC and WAXS signals of PEA 46. **Left**: DSC curve from a first heating scan at 20 °C/min. **Right**: WAXS diffraction profiles collected at room temperature from (a) a compression molded sample, (b) a sample annealed 15 min at 150 °C and (c) a sample melt crystallized at 10 °C/min. The only purpose of Figure 2 is to establish the nomenclature used in this work: the source of the DSC and WAXS data and further details are discussed in Section 3.2, Section 3.3, Section 3.4, Section 3.5, Section 3.6 and Section 3.7.

The synthetic method used to obtain PEA 46 and its polymeric structure are shown in Figure 1. In the literature [27], PEA 46 showcased an interesting behavior during Differential Scanning Calorimetry (DSC) analyses, particularly in the temperature range between 140 and 190 °C, where several consecutive thermal transitions were observed. These phenomena and the corresponding changes in the structure of PEA 46 required a more specific study to be better understood, so with the present work it was decided to carry out a deeper structural characterization of the compression molded material. The DSC results are reported in Table 1, with the exclusion of data regarding the glass transition of PEA 46, which was found at 23 °C during first DSC heating scans carried out at 20 °C/min. For ease of identification, the peaks were labeled I, C1, M1, M2, C3 and M3, and they are attributed in Figure 2, left panel. The only purpose of Figure 2 is to establish the nomenclature used in this work: further information on the corresponding data is discussed in Section 3.

**Figure 3:**
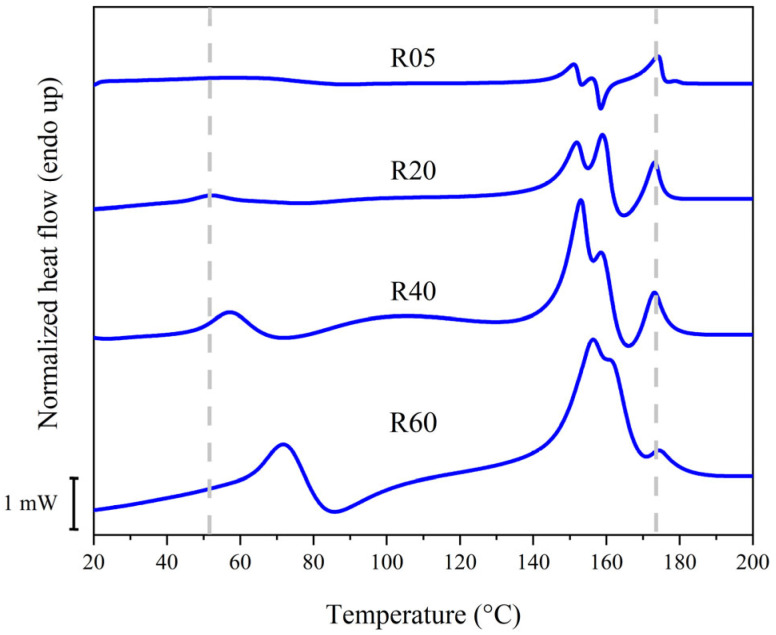
DSC curves from the first heating scan on compression molded samples of PEA 46, at different heating rates, expressed in °C/min.

Peak I was endothermic and located at 52 °C during the first heating scan. By subjecting the sample to DSC experiments at heating rates of 5, 20, 40 and 60 °C/min, it was observed that the position of this peak shifted, depending on the heating rate used (Table 1 and Figure 3). As expected, the increasing heating rate lead to the suppression of the melt-recrystallization. On the contrary, the dependence of the heating rate on the melting temperature could be either positive or negative. By increasing the heating rate, the melting temperature can: (1) decrease, for lamellar thickening (crystal perfectioning); (2) increase, due to superheating and thermal lag [34,35,36,37]. However, for furan-based polymers, this behavior can often indicate that the thermal phenomenon is a second-order transition, like a glass-to-rubber transition, or like the isotropization of a partially ordered mesomorph phase: in fact, mesomorph phases have been observed in the literature on furan-based polyesters, where they are based on hydrogen bonds and π-π stacking between furan rings [20,38]. In particular, peaks M1 and M2 showed smaller changes of their position, indicating that they might not be the simple melting of a crystalline phase: instead, for example, they could be the result of multiple superimposed thermal phenomena or polymorphic thermal transitions. The position of peak M3 remained unchanged, suggesting that it might be originated by the simple first-order melting transition of a crystal. Also, the enthalpy of peak M3 decreased with increasing heating rates: it is reasonable to infer that peak M3 could be originated by the crystals formed by melt-recrystallization. Overall, peak M1 was located at 152 °C during the first heating scan at 20 °C/min, followed by peak M2 at 159 °C, C3 at 165 °C and M3 at 173 °C. Moreover, a wide exothermic depression called C1 was observed, with a minimum at 76 °C and extremes between 58 and 134 °C, approximately. The compression-molded samples of PEA 46 were also subjected to preliminary WAXS analyses, and the samples only showed a single reflection at a 2θ value equal to 20.2°, the same found in the literature on as-synthesized samples [27,39].

**Figure 4:**
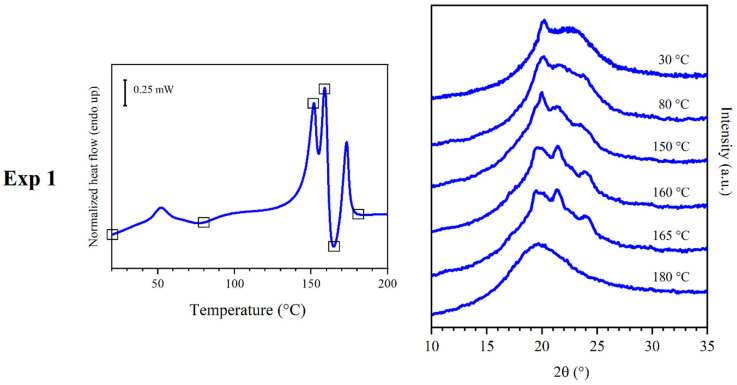
DSC curves with a heating rate of 20 °C (**left**) and WAXS traces (**right**) obtained from experiment 1. WAXS traces were obtained at the temperature represented by squares on the DSC curves.

**Figure 5:**
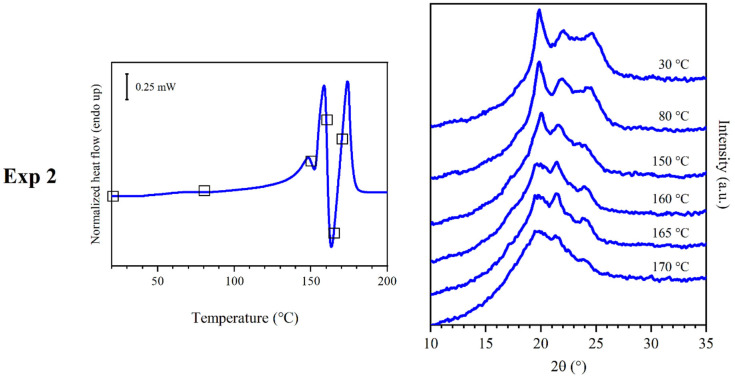
DSC curves (**left**) and WAXS traces (**right**) obtained from experiment 2. WAXS traces were obtained at the temperature represented by squares on the DSC curves.

**Figure 6:**
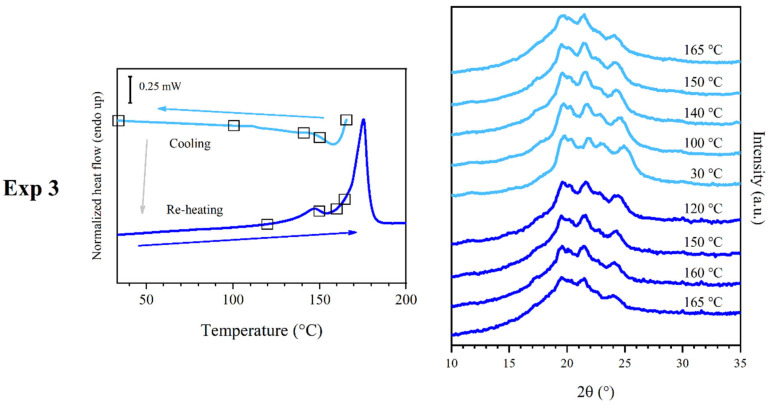
DSC curves (**left**) and WAXS traces (**right**) obtained from experiment 3. WAXS traces were obtained at the temperature represented by squares on the DSC curves. Cooling and re-heating were carried out at 10 and 20 °C/min, respectively.

Considering the results that have been reported thus far, the investigation of the crystal structure of PEA 46 was organized in three experiments, which will be discussed individually. They are represented in Figure 4, Figure 5 and Figure 6 and the findings are summarized in Table 2 and Table 3. In Table 3 and Figure 2, reflections were labeled as R0, R1, R2, R3, R4 and R5 to connect the plots of Figure 2, with the angular data in Table 3.

### 3.2. Results of Experiment 1

In experiment 1 (Figure 4), a compression molded sample of PEA 46 was subjected to in situ WAXS analyses, in order to have a general understanding of the set of phenomena under investigation. The analyses were carried out throughout a temperature scan that was set to be as similar as possible to the one of a DSC scan previously performed at a heating rate of 20 °C/min. At 30 °C, the conditions were the same as the ones in which the WAXS analyses were carried out in the literature [27] and the diffraction pattern was also similar, with the unique reflection at angular position 2θ = 20.2° over a bell-shaped profile. At 80 °C, temperature higher than DSC peak I and roughly at the center of exothermic process C1, two other reflections shaped above the amorphous halo, at 21.6 and 23.7 °C, respectively. At 150 °C, a WAXS analysis was carried out in correspondence with peak M1. In spite of M1 being identified as a melting peak, the WAXS scan showed increased crystallinity, since the reflections further increased their intensity. At 160 °C, another WAXS analysis was carried out in correspondence with peak M2, and once again, the crystalline structure did not seem to be disrupted by the endothermic phenomenon, but the pattern underwent further changes, showing one additional reflection at 19.5 °C. At 165 °C a WAXS scan was carried out in correspondence of peak C3: the profile did not show significant variations, apart from a general increase in reflection intensity. Finally, a WAXS analysis was carried out at 180 °C, above peak M3, and since no diffraction peaks were detected, the material was confirmed to be isotropic. In order to better understand the phases, which were observed at 150 °C and 165 °C, experiments 2 and 3 were carried out.

### 3.3. Results of Experiment 2

Experiment 2 (Figure 5) was carried out in order to better understand the phases, which were observed at 150 °C in experiment 1. A compression molded film of PEA 46 was annealed for 15 min at 150 °C inside an oven. Afterwards, a sample of the annealed film was subjected to DSC scans (at 20 °C/min) and WAXS analyses during a temperature scan (also at 20 °C/min). The DSC results showed that peak I and C1 disappeared, while peak M1 significantly reduced its melting enthalpy, which decreased from 23 to 3 J/g. The melting enthalpy of M2 and C3 remained approximately similar, while the one of M3 increased from 7 to 17 J/g. The corresponding WAXS diffraction profile is reminiscent of the one recorded at 150 °C in experiment 1. The main differences were (i) peak intensity, since the sample underwent annealing treatment; (ii) the shift of reflections towards higher angles, because the temperature during the WAXS scan was lower. WAXS scans were also performed during heating, throughout a temperature scan, which was programmed to be as similar as possible to the one of a DSC scan at a heating rate of 20 °C/min.

### 3.4. Results of Experiment 3

Experiment 2 (Figure 6) was carried out in order to better understand the phases which were observed at 165 °C in experiment 1. A compression molded sample of PEA 46 was positioned in the temperature control device of the diffractometer, quickly heated to 165 °C, held at 165 °C for 60 s, then cooled at 10 °C/min to 30 °C. Several WAXS analyses were performed in situ throughout the cooling scan. Before cooling, at 165 °C the profile was found to be similar to that collected in experiment 1 at the same temperature. In the scans performed at 150, 140, 100 and 30 °C the same reflections were observed, with minimal variations in their position, but with increasingly higher intensity. This temperature treatment was reproduced with a DSC analysis, in which a sample was subjected to the same heating and cooling treatment. Additionally, one re-heating scan was performed at 20 °C/min, to verify the structure obtained from the cooling scan at 10 °C/min. The re-heating scan, after heating and cooling, was performed during both DSC and WAXS analyses, under the same conditions. The cooling scan showed an exothermic crystallization peak corresponding to C3, with maximum at 158 °C and enthalpy equal to 31 J/g, distributed over a remarkably high temperature range, approximately from 165 to 80 °C. The following heating scan showed two peaks. The one at 147 °C was identified as peak M1 and had very low melting enthalpy, equal to 2 J/g, while the one at 175 °C was identified as peak M3 and had a melting enthalpy equal to 38 J/g, similar to the melt crystallization enthalpy recorded for peak C3 during the cooling scan.

### 3.5. Discussion of Experiment 1

During experiment 1 (Figure 4), several ordered phases could be observed. At 30 °C, the WAXS pattern is very simple: it shows a peak corresponding to 0.44 nm distance and a wide bell-shaped band due to disorder of the main part of the polymer chain. This pattern can originate from a very low amount of crystalline phase with a low degree of order in an otherwise amorphous material, or from a mesophase. The strong shift in the position of peak I in DSC experiments (Figure 3) verified the latter hypothesis. For this reason, the presence of DSC endothermic peak I and subsequent exothermic conversion C1 should be the evolution of a mesophase. Chains should gain mobility at peak I and partially develop a crystal phase with low degree of crystallinity and with crystals that have a low degree of order. In fact, the diffraction profiles recorded at 80 °C showed broad reflections, indicating imperfect crystals. The WAXS pattern collected at 150 °C displayed reflections of higher intensity. For this reason, it is possible that a crystallization phenomenon, which will be called C2, could be superimposed to melting peak M1, and the sum of the two resulted in a neat endothermic peak.

### 3.6. Discussion of Experiment 2

To understand the behavior of the material better, it was deemed necessary to subject the sample to annealing, to obtain a clear improvement of the clarity of the diffraction profile by enhancing its crystallinity. This was achieved through experiment 2. Here, the crystalline phase obtained after annealing at 150 °C will be called phase α, while in a state of lower order (as in experiment 1) will be called α′. Phase α could be considered to be reminiscent of the folded hydrogen-bonded α sheet found in even-even polyamides [27,40]. Although some diffraction peaks were found at distances similar to the ones found in the literature for polyamides with more regular repeating units, the presence of different secondary amido diols in PEA 46 is expected to lead to an overall different WAXS pattern, which is insufficient to determine a specific isomorphous structure. Regardless, it might be possible that in the limited volume of ordered portions the chains may adopt a primarily planar zig-zag conformation with lateral packing similar to other polymers [40]. The unit cells and the crystal structure of semi-aromatic polyamides and FDCA-based furanic-aliphatic polyamides have not been investigated yet, and will require to be studied in detail in the future. At 160 °C, once again, WAXS analyses did not show a decrease in crystallinity associated with endothermic peak M2, but rather an increase, accompanied by the development of additional reflections. This highlights the presence of a phase different from the previous one, which in this work will be called phase β (or β’ when in a lower degree of order). Similarly to what was observed for melting M1 and crystallization C2, at about 160 °C the melting and recrystallization of the material might have happened. In particular, phase α’ might have melted at M1 and phase β’ might have formed in a superimposed exothermic crystallization. It is hypothesized that part of C3 might have been superimposed with M1/C2, while the rest of the crystallization was visible with a distinct exothermic peak during DSC scans. Regardless of the underlying phenomena which might have contributed to the observation of peaks M1 and M2, it should be noted that the formation of phase α’ should have happened during the DSC heating scan. In fact, in spite of the relatively high total melting enthalpy observed during DSC analyses, PEA 46 showed a low degree of crystallinity during WAXS experiments at room temperature, equal to 5 ± 2%, attributed to the single wide reflection at 20°, associated with peak I. At 165 °C, WAXS analyses showed almost no variation in intensity and position of the crystalline reflections, confirming that the crystallization of phase β’ was complete. Finally, PEA 46 was found to be isotropic during WAXS analyses at 180 °C, demonstrating that endothermic DSC peak M3 was associated with the melting of phase β’. During experiment 2 (Figure 5), the brief annealing treatment at a temperature around recrystallization C2 was found to be effective in enhancing phase α, proving that the formation of this phase should be favored by reaching 150 °C. In fact, DSC peak M1 was found to have considerably lower enthalpy than peak M2: the cold crystallization of phase α was favored over the formation of the mesophase or phase α’, which may be considered as less ordered versions of the same structure. As a consequence, the melting of phase α prevailed. As further proof, reflection R1 was found to be particularly intense during WAXS analyses. The WAXS scans performing during heating confirmed that phase α started melting around 150 °C, and phase β formed before the complete isotropization of the material. It should be noted that all reflections shown in the annealed sample had 2θ values slightly shifted from the ones observed during experiment 1, because of the different temperature at which the WAXS data were acquired.

### 3.7. Discussion of Experiment 3

Experiment 3 (Figure 6) showed that the cooling treatment was effective at enhancing phase β’, to the point that, for the sake of simplicity, the final phase observed during WAXS at 30 °C was called phase β, to distinguish it from its version with lower degree of order, phase β’. The crystallization phenomenon observed during the cooling DSC scan was centered in the same position as peak C3, confirming that the crystallization of phase β should happen at about 158 °C. It was also interesting to note that the crystallization was particularly extended over a wide range of temperature, in spite of the slow cooling rate. This observation could support the claim that C3 is partially superimposed with M2 and other peaks during heating DSC scans. The re-heating scan performed for experiment 3 confirmed every other hypothesis by showing the presence of peak M3 with high melting enthalpy and only a small peak M1, which might indicate the possible presence of traces of phase α’. However, the WAXS scans associated with the re-heating of the sample showed that the crystal phase evolved in the same way it did during cooling, without showing the presence of phase α’. The additional WAXS reflection of phase β at 22.8 °C could be attributed to the lower intensity of the signals in the case of the diffraction profiles of phase β’ collected during in situ scans, which made this reflection not intense enough to emerge from the amorphous halo and instrumental noise.

### 3.8. Overview

**Figure 7:**
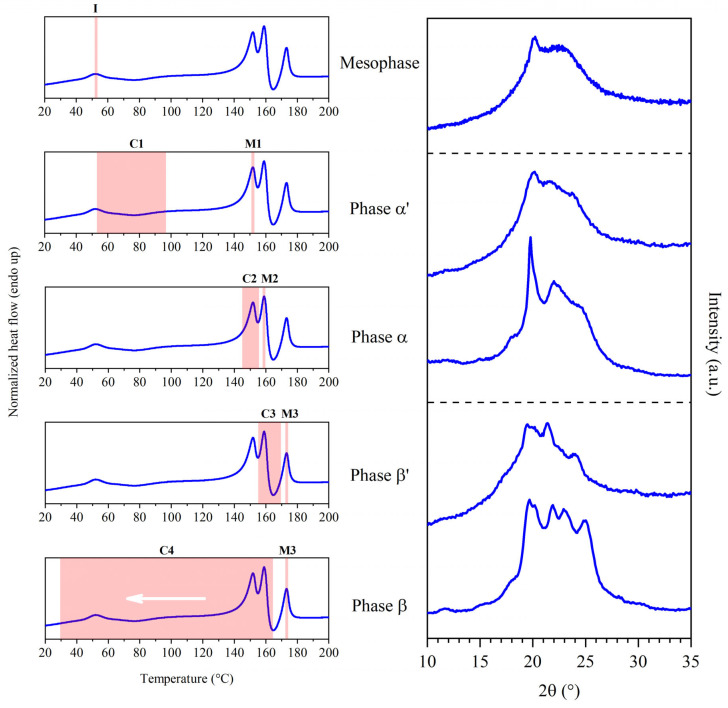
Final interpretation of the DSC transitions observed at 20 °C/min. On the same DSC curve (**left**) are highlighted melting and crystallization phenomena (pink) and their corresponding WAXS profiles (**right**). I, C and M stand for isotropization, crystallization and melting, respectively. WAXS scans of mesophase, phase α, phase β in this figure were collected at room temperature. Crystallization C4 took place after melting, during a cooling scan.

Discussing the data obtained from DSC scans at different heating rates (Table 1 and Figure 3) was found to be useful to further support the overall interpretation of the experimental results. Among the DSC data at varying heating rate, there does not seem to be a direct correlation between the cold crystallization enthalpy of C1 and the melting enthalpy of M1. It is possible that peak C1 might be partially superimposed with peak I, leading to an underestimation of its actual crystallization enthalpy. This is in accordance with the hypothesis that peak I and C1 could represent a sequence of isotropization and recrystallization of the mesophase. Another possibility to explain the incongruence between the enthalpies of peak C1 and M1 is that multiple melting and recrystallization phenomena might be superimposed under peak M1, resulting in the endothermic peak M1. As shown in DSC scans at different heating rates, peak C3 and M3 had about the same crystallization and melting enthalpy at heating rates of 20 and 40 °C/min, confirming that phase β should have been originated by crystallization C3. The correspondence between the enthalpies of peak C3 and M3 was not found to be always perfect, such as in the case of a heating rate of 5 and 60 °C/min. In these cases, the cold crystallization enthalpy of C3 is lower than the melting enthalpy of M3. This could be attributed to the potential partial superimposition of peak C3 with other peaks, such as peak M2, which would result in an underestimation of the actual crystallization enthalpy of C3. In order to have a more effective overview of the results of this work, the main findings will be summarized. A visual summary can also be found in Figure 7.

▪ Mesophase: this phase formed after compression molding. It underwent isotropization at I and showed the WAXS pattern reported in Figure 2a.
▪ Phase α’ (phase α with low crystallinity): this phase crystallized at C1 and might have undergone multiple melting and recrystallization phenomena leading to the conversion into phase α. Its pattern was recorded in situ during the heating process.
▪ Phase α: this phase crystallized at C2 and melted at M1, showing the WAXS pattern reported in Figure 2b; the peaks were sharper than the ones found in phase α’. Crystallization C2 was likely superimposed to melting M1.
▪ Phase β’ (phase β with low crystallinity): this phase melted at M2; its diffraction pattern was recorded in situ. Crystallization C3 was likely partly superimposed to melting M2.
▪ Phase β: this phase was obtained by favoring the crystallization of the sample via melt crystallization, over a temperature range corresponding to C4, and it melted at M3, showing the WAXS pattern reported in Figure 2c. It should effectively have the same structure as phase β’, but with enhanced degree of crystallinity.

## 4. Conclusions

This initial study on the microstructure of PEA 46 had the objective of exploring the complex series of DSC transitions found in compression molded samples of this polymeric material. For this reason, film samples of PEA 46 were analyzed by means of DSC and WAXS experiments. The evidence allowed the identification of six distinct WAXS reflections: R0 (18.2°), R1 (19.5 ÷ 20.1°), R2 (19.7 ÷ 20.2°), R3 (21.4 ÷ 22.0°), R4 (22.7 ÷ 22.8°), R5 (23.5 ÷ 24.6°). These reflections appeared in three different phases: firstly, a mesophase was the only phase observed at room temperature. After a series of partially superimposed melting and recrystallization phenomena, the temperature scan during DSC lead to the formation of phase α’ (melting at 148 ÷ 152 °C) and phase α (melting at 158 ÷ 159 °C), which were found to have the same crystalline structure, but increasing degree of order. Phase α was hypothesized to be formed by α-type crystals, similar to the ones found in even-even polyamides. An additional melting and recrystallization phenomenon lead to the formation of phase β, which melted at 173 ÷ 174 °C, making the sample completely amorphous and isotropic. Phase α could be enhanced with a thermal annealing treatment, while phase β could be enhanced with a melt crystallization treatment. Overall, the cross-comparison of DSC results with temperature-dependent WAXS measurements successfully provided key structural information corresponding to the thermal transitions of PEA 46. Outside of the scope of this initial, exploratory study, other experimental methods will be useful to characterize the complex polymeric phases discovered in this paper. Specifically, in the future, experimental work could be focused on the theorized superimposition of peaks, which could be deconvoluted by means of MT-DSC analyses. Also, a Flash DSC experiment could be useful to assess the potential presence of phenomena with kinetics incompatible with the heating rates used in this work. The presence of a mesomorph phase might be verified with SAXS experiments. Finally, 3D modeling and simulations could assist in the determination of the type and composition of the crystalline cells under study.

## Figures and Tables

**Table 1:** DSC data from the first heating scan on compression molded samples of PEA 46, at different heating rates, expressed in °C/min. * The melting temperature and melting enthalpy could not be determined.

Label	I	C1	M1	M2	C3	M3
5 °C/min						
T (°C)	ND *	88	151	156	158	173
ΔH (J/g)	ND *	24	23	3	14	30
20 °C/min						
T (°C)	52	76	152	159	165	173
ΔH (J/g)	9	4	23	15	6	7
40 °C/min						
T (°C)	57	75	153	159	166	173
ΔH (J/g)	5	6	31	8	4	4
60 °C/min						
T (°C)	72	86	156	162	-	173
ΔH (J/g)	6	16	22	10	-	1

**Table 2:** DSC data of experiments 1, 2 and 3.

Label		I	C1	M1	M2	C3	M3
Experiment 1							
Heating	T (°C)	52	76	152	159	165	173
	ΔH (J/g)	9	4	23	15	6	7
Experiment 2							
Heating	T (°C)	-	-	148	158	163	174
	ΔH (J/g)	-	-	3	13	9	17
Experiment 3							
Cooling	T (°C)	-	-	-	-	158	-
	ΔH (J/g)					31	
Re-heating	T (°C)	-	-	147	-	-	175
	ΔH (J/g)	-	-	2	-	-	38

**Table 3:** WAXS reflections of experiments 1, 2, 3 and degree of crystallinity (X_c_) for each WAXS analysis. * Scan collected above room temperature; peak positions were affected by thermal expansion.

Label T (°C)	R0 2θ (°)	R1 2θ (°)	R2 2θ (°)	R3 2θ (°)	R4 2θ (°)	R5 2θ (°)	X_c_ (%)
Experiment 1							
30	-	-	20.2	-	-	-	5 ± 2
80 *	-	-	20.1	21.6	-	23.7	7 ± 2
150 *	-	-	20.0	21.4	-	23.5	8 ± 2
160 *	-	19.5	20.0	21.4	-	23.9	10 ± 1
165 *	-	19.5	20.1	21.4	-	24.0	10 ± 1
180 *	-	-	-	-	-	-	0
Experiment 2							
30	18.2	19.8	-	22.0	-	24.6	18 ± 1
80 *	18.2	19.8	-	21.9	-	24.3	18 ± 1
150 *	-	20.1	-	21.6	-	23.9	16 ± 1
160 *	-	19.6	20.0	21.4	-	23.9	15 ± 1
165 *	-	19.5	20.1	21.4	-	23.9	15 ± 1
170 *	-	19.7	19.7	21.4	-	23.8	13 ± 1
Experiment 3							
Cooling							
165 *	-	19.5	20.0	21.5	22.8	24.0	9 ± 2
150 *	-	19.5	20.1	21.5	22.7	24.2	12 ± 1
140 *	-	19.6	20.2	21.6	22.8	24.3	13 ± 1
100 *	-	19.7	20.2	21.7	22.9	24.5	14 ± 1
30	-	19.8	20.4	21.8	23.0	24.9	15 ± 1
Re-heating							
120 *	-	19.6	20.2	21.6	22.8	24.3	14 ± 1
150 *	-	19.5	20.2	21.5	22.7	24.2	13 ± 1
160 *	-	19.5	20.1	21.4	22.6	24.1	12 ± 1
165 *	-	19.5	20.1	21.4	22.6	24.0	10 ± 1

## Data Availability

The original contributions presented in this study are included in the article. Further inquiries can be directed to the corresponding author.
